# Supplementary material for: Harnessing Avidity: Quantifying the Entropic and Energetic Effects of Linker Length and Rigidity for Multivalent Binding of Antibodies to HIV-1
Source: Cell Syst. 2019 Nov 27;9(5):466–474.e7. doi: 10.1016/j.cels.2019.09.007 (PMC6892280; doi:10.1016/j.cels.2019.09.007)
Supplement: Document S1. Figures S1–S6 [file mmc1.pdf]

Cell Systems, Volume *9*

Supplemental Information

Harnessing Avidity: Quantifying the Entropic and Energetic Effects of Linker Length and Rigidity for Multivalent Binding of Antibodies to HIV-1

Tal Einav, Shahrzad Yazdi, Aaron Coey, Pamela J. Bjorkman, and Rob Phillips

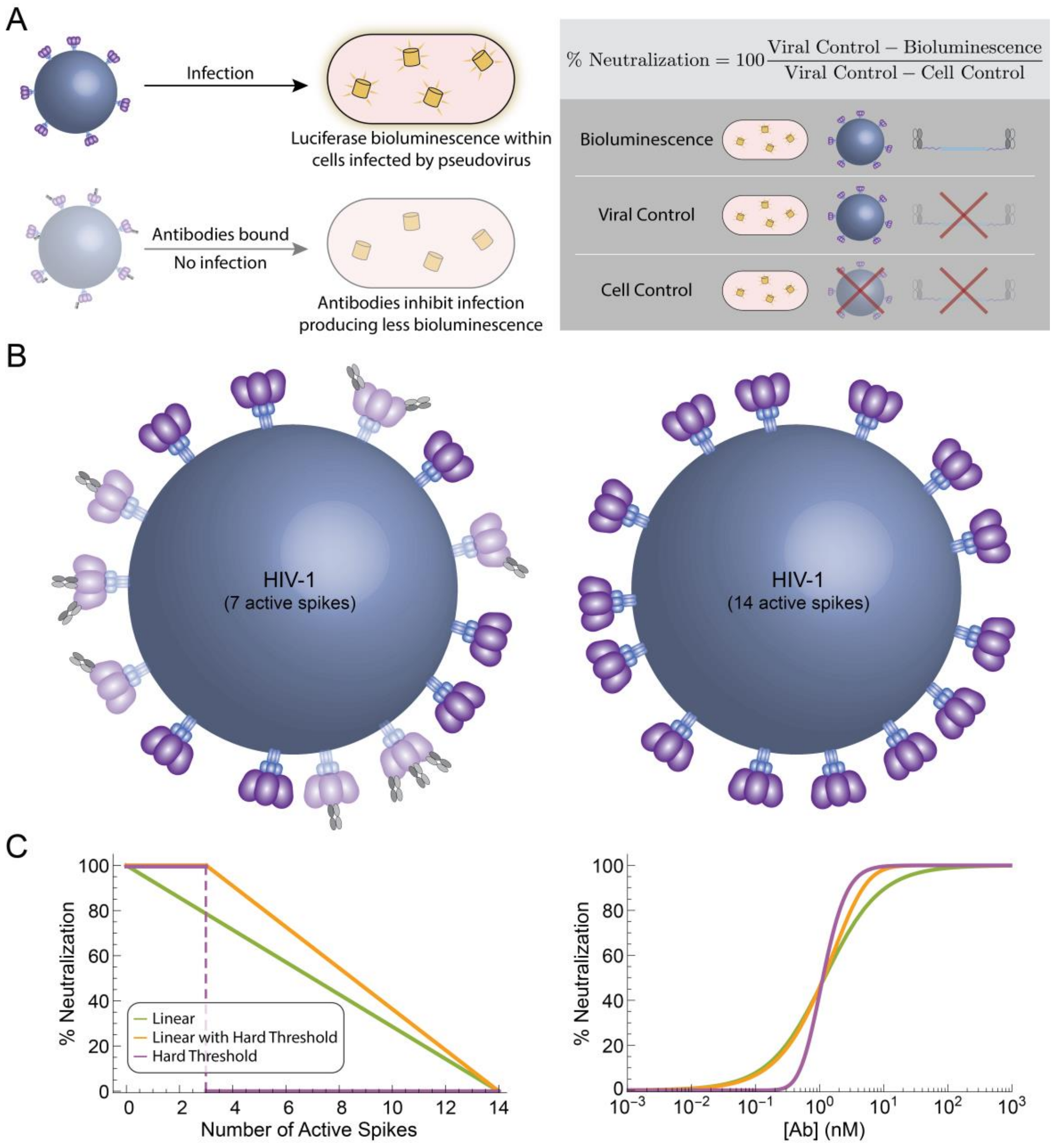

Figure S1. A Linear model of HIV-1 neutralization, related to STAR Methods. (A) In previous work, neutralization of HIV-1 pseudovirus was assayed by evaluating reporter cells that emit light upon HIV-1 infection via luciferase (Galimidi et al., 2015). Adding an antibody results in decreased bioluminescence. (B) We model each HIV-1 virion as having 14 spikes that are inactivated (represented as partially transparent spikes) when a Fab is bound to any of the three binding sites on an Env trimer. In the main text, we assume a linear model in which the infectivity of a virion is proportional to its number of active spikes; for example, the virus on the right will be twice as infective as the virus on the left. (C) Comparison of different models for % neutralization (or the relative infectivity defined as $100 - \%$ neutralization) as a function of the number of active (unbound) HIV-1 spikes (left) or antibody concentration (right). The geometric factor α in Eq. 4 quantifying the effects of diFab avidity was adjusted for each model (1.5×10^6 for the linear model; 10^6 for the linear model with a hard threshold; 7×10^6 for the hard threshold model) to match their IC_{50}s. The values of the remaining parameters were the same as in Fig. 2.

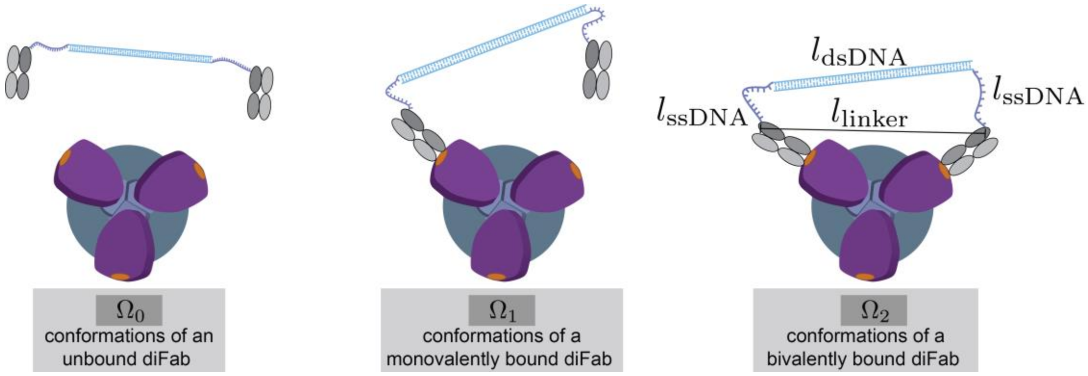

Figure S2. Conformational states of a diFab, related to STAR Methods. We denote the number of microstates Ω_j of a diFab bound with j Fabs. To bivalently bind, the linker region of a diFab (a length l_{dsDNA} of dsDNA flanked by two lengths l_{ssDNA} of ssDNA) must span a distance l_{linker} that is dictated by the Env's structure. The dsDNA is a rigid rod while the ssDNA is modeled as an ideal chain.

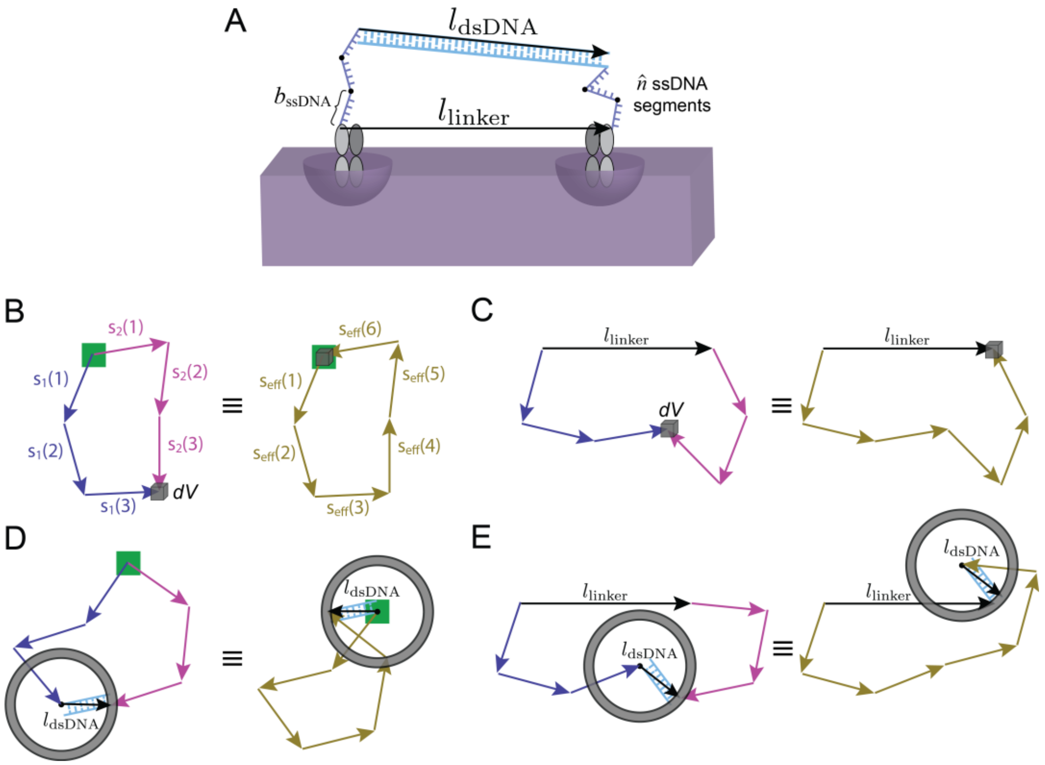

Figure S3. The ideal chain model for a diFab linker, related to STAR Methods. (A) We compute the probability $p(l_{\text{dsDNA}}, l_{\text{ssDNA}}, l_{\text{linker}})$ that the ssDNA ($\hat{n}$ segments of length b_{ssDNA}) and dsDNA (1 rigid segment of length l_{dsDNA}) in the diFab linker will end at the appropriate distance $\vec{l}_{\text{linker}}$ required for bivalent binding. We consider the following cases: (B) $\vec{l}_{\text{dsDNA}} = \vec{0}, \vec{l}_{\text{linker}} = \vec{0}$; (C) $\vec{l}_{\text{dsDNA}} = \vec{0}, \vec{l}_{\text{linker}} \neq \vec{0}$; (D) $\vec{l}_{\text{dsDNA}} \neq \vec{0}, \vec{l}_{\text{linker}} = \vec{0}$; and the full model (E) $\vec{l}_{\text{dsDNA}} \neq \vec{0}, \vec{l}_{\text{linker}} \neq \vec{0}$. The two random walks start at each end of $\vec{l}_{\text{linker}}$, which is denoted by a green square when $\vec{l}_{\text{linker}} = \vec{0}$ (Panels B and D). Random walks end in the same location if they are within a small volume dV of each other (Panels B and C) or if their ends are within dV of the dsDNA of length l_{dsDNA} (Panels D and E). In each case, the two ssDNA random walks are combined into a single effective random walk with $2\hat{n}$ steps.

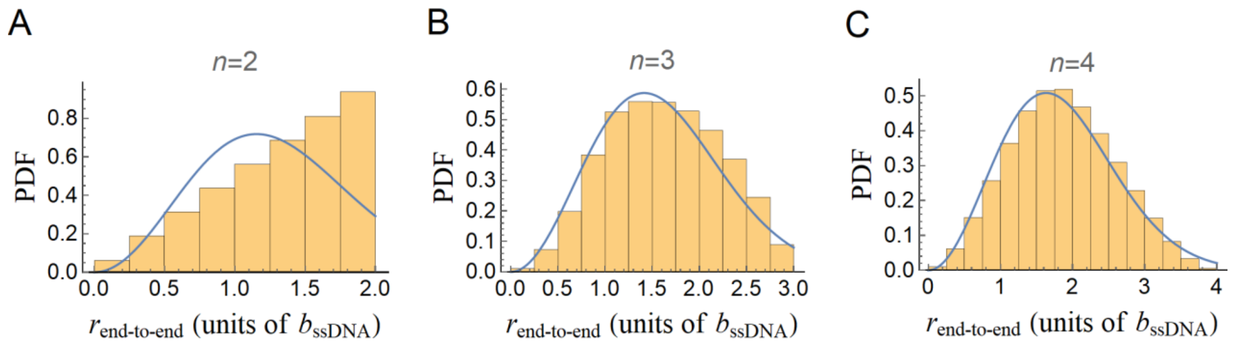

Figure S4. The ideal chain model applies for ≥ 3 segments, related to STAR Methods. Comparing the analytic form of the ideal chain model in the large $\hat{n}$ limit (Eq. 14) with numerical simulations of a 3D random walk with (A) $\hat{n} = 2$, (B) $\hat{n} = 3$, and (C) $\hat{n} = 4$ segments. The y-axis shows the probability density function of the trials. As few as three segments are sufficient to closely match the large $\hat{n}$ limit.

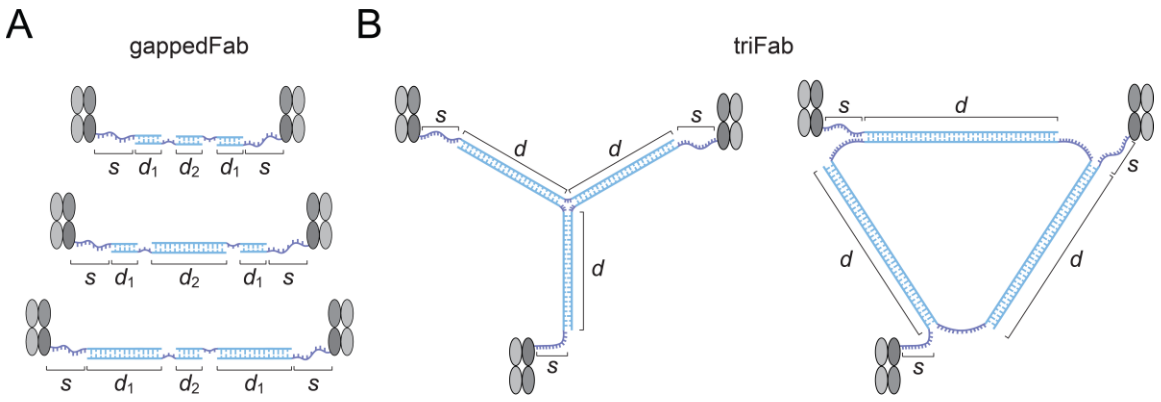

Figure S5. Generalizing the model to other DNA-Fab designs, related to STAR Methods. (A) GappedFabs have ssDNA break in their dsDNA, providing flexibility while keeping each segment rigid. (B) Two alternative triFabs designs (different from the triFab design in Fig. 1B) composed of three Fabs connected by DNA.

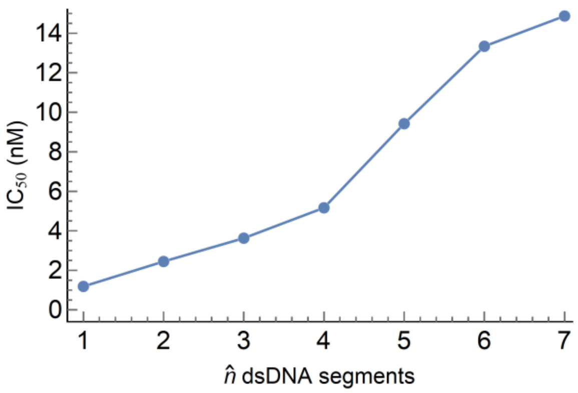

Figure S6. Potency of a gapped diFab made up of 62 bp dsDNA broken into $\hat{n}$ equal length pieces, related to STAR Methods. Breaking up the dsDNA increases the entropic cost of bivalent binding, decreasing its potency and increasing its IC_{50}.
